# A mixture of postbiotics/tyndallized probiotics reduces trimethylamine (TMA) in trimethylaminuria models: Evidence from *in vitro* and *in vivo* studies

**DOI:** 10.3389/fphar.2025.1591825

**Published:** 2025-10-08

**Authors:** Giuseppe Giannini, Sara Soldi, Marina Elli, Valeria Sagheddu, Andrea Castagnetti, Elisa Viciani, Laura Salvini, Gianfranco Battistuzzi, Ferdinando Maria Milazzo, Simona Alibrandi, Antonina Sidoti

**Affiliations:** ^1^ R&D Alfasigma SpA, Roma, Italy; ^2^ AAT—Advanced Analytical Technologies Srl, Piacenza, Italy; ^3^ Wellmicro srl, Bologna, Italy; ^4^ TLS - Fondazione Toscana Life Science, Siena, Italy; ^5^ Department of Biomedical and Dental Sciences and Morphofunctional Imaging, University of Messina, Messina, Italy; ^6^ Department of Biomolecular strategies, Genetics, Cutting-Edge Therapies, I.E.ME.S.T., Palermo, Italy

**Keywords:** TMA, TMAU, TMAO, CVD, postbiotic, rare disease, tyndallized

## Abstract

**Introduction:**

Trimethylaminuria (TMAU), also known as “fish-odor syndrome,” is a rare metabolic disorder characterized by a body malodor that smells like a decaying fish. This syndrome is caused by a FMO3 liver enzyme malfunction, leading to trimethylamine (TMA) accumulation. To date, there is no definitive therapeutic treatment but only palliative care for TMAU, such as a controlled diet, taking antibiotics, or using acidic soaps to capture sweat-released TMA.

**Methods:**

Here, we describe an innovative approach for the treatment of this disorder, where the use of postbiotics/tyndallized probiotics is able to effectively inhibit the bacterial TMA lyase present, thus preventing the formation of TMA. We obtained a preparation (a mixture of tyndallized probiotics and their postbiotics) that was derived from the fermentation of *Lacticaseibacillus paracasei* in the presence of garlic extract and senna leaf. This preparation was used in *in vitro* assays on human fecal slurry while monitoring the levels of TMA released over time, and it was also tested *in vivo* in both *Mus musculus* C57BL/6 (FMO3^+/+^) strain WT and C57BL/6^-Fmo3em1Smoc^ (KO) mouse models to measure the trimethylamine N-oxide (TMAO) and TMA levels in the blood and urine, along with gut microbiota analysis in feces via next-generation sequencing (NGS).

**Results:**

*L. paracasei* fermentation yielded 4.1 × 10^12^ CFU/g lyophilized powder. *In vitro* assays involving fecal slurries supplemented with the fermentation product demonstrated a reduction in TMA levels, and the NGS analysis revealed that *Collinsella*, *Clostridium*, and *Streptococcus* were the most common bacterial genera that produced TMA. The *in vivo* study showed a significant reduction in TMAO levels in C57BL/6(FMO3^+/+^) strain WT mouse models and in TMA levels in C57BL/6^-Fmo3em1Smoc^ (KO) mouse models. In addition, bacteria belonging to the TMA-producing genera were still present after treatment with the tested compounds, excluding their bactericidal action. The postbiotics obtained may find a useful therapeutic application both in the prevention of cardiovascular events and as valid supports to reduce TMA production in TMAU patients.

## 1 Introduction

### 1.1 Relationship among gut microbiota, TMA and TMAO production, and human diseases

Consolidated literature from over the last 20 years confirms the relationship between intestinal microbiota and a series of diseases (Chenhuichen et al., 2022; Sorboni et al., 2022). These include cardiovascular diseases (CVDs) and their dependence on a metabolite, trimethylamine N-oxide (TMAO). Indeed, elevated plasma TMAO levels may increase the risk of atherosclerotic cardiovascular disease (ASCVD) (Yang et al., 2019; Sanchez-Rodriguez et al., 2020; Lee et al., 2021). Plasma trimethylamine (TMA) levels are ten-fold lower than the TMAO levels under normal physiological conditions. However, TMA but not TMAO has been shown to be associated with severe aortic stenosis and gestational diabetes risk (Huo et al., 2019). In addition, an excess of TMA can be concerning because being very volatile and foul-smelling, it creates great discomfort. Moreover, its ability to overcome the blood–brain barrier allows this molecule to exert some neurological toxic effects. High levels of TMA in the blood have been associated with cognitive deterioration, which is perhaps due to the direct damaging action of TMA on the cerebrovascular endothelium (Hoyles et al., 2021). The gut microbiota plays an important role in the synthesis of these two molecules, particularly TMA. TMAO production occurs mainly by the liver flavin-containing monooxygenase 3 (FMO3) enzyme.

### 1.2 Biochemical mechanisms of TMA production

Most of the TMA is synthesized in the intestine (colon) by the action of some anaerobic commensal gut bacteria containing choline- (or carnitine-) TMA lyase, an enzyme consisting of two units: a catalytic unit (CutC) and its activating enzyme (CutD) (Bodea and Balskus, 2018). This enzyme metabolizes some dietary ingredients, such as choline, and other choline-containing compounds (phosphatidylcholine/lecithin, phosphocholine, sphingomyelin, *etc*.), betaine, and L-carnitine and its metabolite γ-butyrobetaine (GBB), releasing TMA after the cleavage of the carbon (C)–nitrogen (N) bond of choline/carnitine (Zhu et al., 2014). Over 20 bacterial species contribute to TMA production (Ramireddy et al., 2021; Sidoti et al., 2024). Absorbed TMA is converted to TMAO primarily by hepatic FMO3 (>10× more efficient than FMO1). Humans express five FMO isoforms (FMO1–5), but FMO3 is the dominant hepatic enzyme responsible for detoxifying TMA into odorless TMAO (Lawton et al., 1994; Janeiro et al., 2018).

### 1.3 Trimethylaminuria (TMAU): etiology, psychosocial impact, and incidence

**FIGURE 1:**
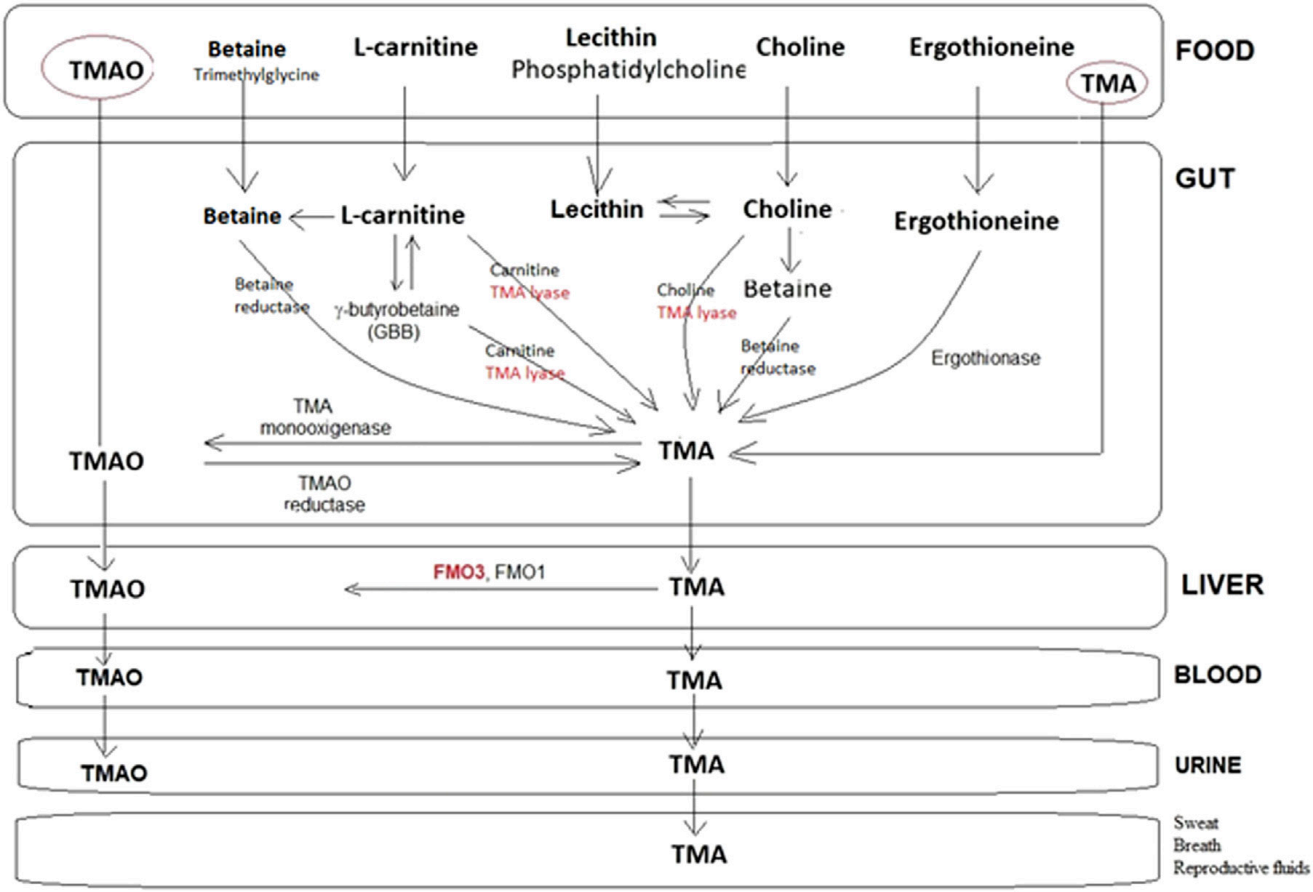
Representative metabolic pathways for the production and metabolism of TMA by the human microbiota. Choline and carnitine can form TMA directly or following conversion to betaine. A few steps share a TMA lyase in the transformation of the main substrates into TMA, which, in turn, is transformed at the liver level into TMAO or eliminated as such with the urine, sweat, breath, *etc*., in the case of FMO3^(-/-)^. Scheme based on the information described in several papers.

Trimethylaminuria (TMAU) is caused by TMA accumulation. The latter is excreted through bodily secretions, including sweat, urine, breath, and reproductive fluids. Consequently, TMAU patients exhibit an unpleasant body and breath odor, giving off a strong body malodor; hence, it is also known as “fish odor syndrome” (Figure 1) (Fennema et al., 2016; Yang et al., 2019). At least two different types of TMAU are recognized.

• The primary TMAU (TMAU 1)

It is a genetic form that is caused by a deficit of the FMO3 enzyme determined by *FMO3* gene mutations: homozygote; heterozygote, whose incidence ranges from 0.5%–1% depending on the ethnicity examined (Messenger et al., 2013); or haplotype conditions that are inherited in an autosomal recessive manner (Dolphin et al., 1997; Alibrandi et al., 2021). More than 50 mutations (https://www.hgmd.cf.ac.uk/ac/index.php) that abolish or severely reduce FMO3 activity have been identified (Shimizu et al., 2014). Hence, both the parents of a patient affected by TMAU 1 are carriers of at least one *FMO3* causative mutation. Once the mutation has been identified, an antenatal diagnosis can also be made (Donato et al., 2021).

• The secondary TMAU (TMAU 2)

It is caused by environmental factors, such as menstruation, transient TMAU in newborns and very young children, and viral hepatitis, or is acquired during adult life as a consequence of permanent factors, such as liver cirrhosis or uremia, but in most cases, this form is caused by microbiota alterations. Choline-rich bile further complicates management of TMAU by providing non-dietary TMA precursors (Zeisel, 2014).

The social impact, both for TMAU 1 and TMAU 2 patients, is devastating and is frequently associated with psychiatric conditions such as depression, anxiety, and behavioral disorders. Although the first case of TMAU was described in the literature around 1970 (Humbert et al., 1970), to date, only a few hundred cases have been reported in the literature, with an estimated incidence of 1:25,000–40,000. The cases are decidedly higher for the heterozygous form, where the incidence ranges from a rate of 1% within the white British population to 11% in other ethnic groups, such as the New Guinean population. There is evidence that Afro-American women are particularly affected (Schmidt and Leroux, 2020).

### 1.4 Current treatments and emerging therapeutic strategies

Currently, the main treatment procedure is based on dietary restriction of TMA precursors (Sikand and Severson, 2020) and frequent washing using acidic soaps. The intake of antibiotics to somehow destroy the intestinal microbiota and the administration of the oligosaccharide lactulose, an osmotic laxative, can be additional methods. It is obvious that these approaches can provide some relief only in secondary, transient forms of TMAU, and they are completely inadequate in primary TMAU cases that need long-term therapies. Some promising approaches include the following:

- Garlic-derived allicin: reduces TMAO in murine models (C57BL/6 mice), suggesting anti-atherosclerotic potential (Hall M., 2018).
- TMA lyase inhibitors: synthetic compounds (e.g., fluoromethylcholine, FMC) that irreversibly inhibit CutC/D (Roberts et al., 2018).
- Probiotics: specific strains can lower TMAO levels (Vasquez et al., 2019; Cantero et al., 2022).

### 1.5 Experimental approach

Here, we report the results of an alternative approach based on the use of postbiotics—preparations of inanimate microorganisms and/or their components, which confer health benefits to the host—as inhibitors of intestinal TMA lyase (Salminen et al., 2021). Postbiotics offer several advantages over probiotics, particularly in terms of stability, safety, and efficacy. We focused this research on postbiotics primarily for two reasons: for patients, the use of probiotics is not always indicated, especially in patients with intestinal alterations (endothelial and microbiota). Postbiotics may be a safer option for people with certain health conditions or sensitivities as they do not carry the risk of introducing live bacteria that could potentially cause adverse effects, such as infections or the transfer of antibiotic resistance genes. From the point of view of industrial development, postbiotics, which are the metabolic byproducts of probiotics, are nonliving and, thus, more stable and have a longer shelf life than live probiotic strains. This makes them easier to formulate into commercial products to store without refrigeration. Through a novel process set up for this project, we generated postbiotics by letting the proprietary strain *Lactobacillus paracasei* LMG-S-26420 ferment for 72 h under anaerobic conditions in the presence of a freeze-dried botanical hydroalcoholic extract of garlic (*Allium sativum*). Heat-treated (tyndallized) biomass and the supernatant (postbiotics) were used for the *in vitro* and *in vivo* tests, and FMC was used as a reference inhibitor (Roberts et al., 2018). The *in vitro* test was performed on a fecal slurry sample, monitoring the formation of TMA for 1, 3, and 12 h, and choline (50 µM) was used as a substrate. This was an adaptation of the protocol described by Neilson and collaborators (Iglesias-Carres et al., 2021).

In our case, having used a medium with poor ingredients, with respect to the protocol described in literature (Iglesias-Carres et al., 2021), the added choline was metabolized as a substrate within the first 3 hours. Prolonged times did not allow correct monitoring of the study as the released TMA, being a volatile substance, was dispersed. TMA titration was performed via HPLC/MS. The *in vivo* study was performed in two phases, first using a wild-type mouse (*Mus musculus*) model, C57BL/6 (FMO3 ^+/+^) female mice, where TMAO levels in the blood were measured 3 h after the administration of the inhibitor and 2 hours after the administration of choline orally. Subsequently, the study was repeated in a knock-out mouse model for the FMO3 ^(−/−)^ gene, C57BL/6^-Fmo3em1Smoc^ (pathogenic germ-free). In this case, the levels of TMA in the blood and urine were measured before and after treatment, in addition to an next-generation sequencing (NGS) analysis of the intestinal microbiota.

The study showed that animals treated with the reference inhibitor, FMC, had a >95% reduction in TMAO levels. In the FMO3 knock-out animal model, the reduction in TMA levels after compound treatment was also high and almost comparable to that obtained from FMC treatment.

## 2 Materials and methods

### 2.1 Materials

All the chemicals and reagents used in this study were obtained from commercial suppliers. De Man–Rogosa–Sharpe (MRS) broth and agar were purchased from BD (Difco, Italy). Maltodextrin, dimethyl sulfoxide (DMSO), choline chloride, and the antibiotics amoxicillin, neomycin, and metronidazole were acquired from Sigma-Aldrich. Sodium hydroxide (NaOH, 1M and 5M), ethyl bromoacetate, formic acid, acetonitrile, and methanol were supplied by Merck (Italy). Hydroalcoholic plant extracts were provided by Botanical Cube Inc. (United State). Cell culture reagents, including Dulbecco’s Modified Eagle Medium (DMEM) and fetal bovine serum (FBS), were obtained from Euroclone (Italy). FMC and an additional batch of choline chloride were sourced from A2B Chem LLC (United State), whereas vancomycin was purchased from Millipore. For cytokine analysis, the Mouse IL-10 ELISA Kit was acquired from Invitrogen, which is a part of Thermo Fisher Scientific (United State). The stable isotopes D9-trimethylamine (D9-TMA) and D9-trimethylamine N-oxide (D9-TMAO) were obtained from Cambridge Isotope Laboratories (United State).

### 2.2 Selection and preparation of plant extracts (matrices)

A list (21) of commercial plant extracts obtained by Botanical Cube Inc. was investigated in this study. The extracts were as follows: M01, turmeric (*Curcuma longa*); M02, green tea (*Camellia sinensis*); M03, *Ginkgo biloba*; M04, grape seed (*Vitis vinifera*); M05, garlic (*A. sativum*); M06, Chinese sage (*Salvia miltiorrhiza*); M07, ginger (*Zingiber officinale*); M08, camu camu (*Myrciaria dubia*); M09, garcinia (Garcinia cambogia); M10, mangosteen garcinia (*Garcinia mangostana*); M11, senna leaves (*Cassia angustifolia*); M12, lotus leaves (*Nelumbo nucifera*); M13, frankincense (*Boswellia carterii serrata*); M14, broccoli sprouts; M15, Scutellaria (*Scutellaria baicalensis Georgi*); M16, green coffee; M17, white beans (*Phaseolus vulgaris*); M18, soybean sprouts (*Phaseolus radiatus*); M19, lemon verbena (*Lippia citriodora*); M20, karkadè (*Hibiscus sabdariffa*); and M21, thorn date seed (*Ziziphus jujuba Mill. var. Spinosa*).

A study of this type was difficult to conduct on 21 extracts, and, at the same time, we could not select a limited number of extracts without a broader initial screening. Therefore, all 21 hydroalcoholic extracts were initially considered for IL-cytokine (data shown in Table 3), in addition to the activity of the postbiotics obtained from the 21 extracts left to ferment on different bacterial strains (data not reported in this manuscript). Although almost all the extracts showed variable activity, a selection criterion was introduced based on their *in vitro* activity (higher activity and lowest standard deviation) and on known information on the use of each plant in the treatment of cardiovascular events. The matrices that were finally selected are as follows: M05, M06, M09, and M11.

These freeze-dried extracts were suspended in a hydroalcoholic solution of EtOH/H_2_O (1:9).

### 2.3 Preparation of postbiotics

The postbiotics were prepared to start from a proprietary strain *Lacticaseibacillus paracasei* (formerly known as *Lactobacillus paracasei*) LMG-S-26420, which was previously characterized for its beneficial features, in the presence of at least one plant matrix. Among the various matrices tested, the hydroalcoholic extracts of garlic (*A. sativum*) and senna leaves (*C. angustifolia*) were finally selected. Different fermentation processes were applied to the lab-scale production of the postbiotics (Giannini et al., 2024).

#### 2.3.1 Fermentation conditions

*L. paracasei* LMG-S-26420 was first sub-cultured in 40 mL of MRS (De Man–Rogosa–Sharpe, BD, Difco, Italy) broth for 18 h at 37 °C under microaerophilic conditions. The inoculum size for the pilot-scale fermentation was 1%. *L. paracasei* LMG-S-26420 was grown in a Sartorius fermenter (Biostat A Plus model) for 24 h at 37 °C, at pH 5.5, and controlled by 5M NaOH. Fermentation was carried out in a final volume of 4 L. The MRS fermentation broth medium chosen was Difco, BD. Four different time points after the inoculum (T0) were sampled, namely, T1.5, T3, T18, and T24 h. At the end of fermentation, the biomass was centrifuged, the supernatant was discarded, and the pellet was resuspended in 100 mL of a solution composed of 20% maltodextrin as a cryoprotectant agent. The pellet was completely resuspended, ensuring dissolution of any clumps, and a viable count was performed. The homogeneous solution of the pellet and cryoprotectant agent was placed in the freeze-drying tray that was pretreated with ethanol. The tray was placed at −80 °C for 18 h. The freeze-dryer BenchTop Pro with Omnitronics was used to freeze-dry the resuspended fermentation product. The automatic freeze-drying mode was set (−46 °C condenser temperature and 100 mT for the vacuum system). The product was freeze-dried for 18 h (Savini et al., 2010).

#### 2.3.2 Postbiotic production

Three distinct fermentation processes, designated as A, B, and C, were evaluated for each previously selected matrix. The resulting postbiotics were nomenclatured based on the specific matrix utilized as the fermentation substrate and the type of process applied, as shown in Supplementary Table 1.

Process A was conducted in two sequential steps. In the first step, the grown biomass was suspended in DMEM (Euroclone, Italy). The second step involved adding this initial suspension (designated as step A1) to a fresh mixture of DMEM and the respective matrix.

In contrast, processes B and C were performed as single-vessel or “one-pot” fermentations. This involved suspending the grown biomass directly in DMEM supplemented with FeSO_4_, MgSO_4_, MnSO_4_, and Tween 80, followed by fermentation with the matrix in the same bioreactor.

The three different processes have been described because they were performed from the initial research phase, with the aim of selecting the best postbiotics, to the final one (including the tyndallized probiotic) to satisfy the industrial developability requirements of the process.

##### 2.3.2.1 Process A

A1 The grown biomass of *L. paracasei* LMG-S-26420 pellet, 2.85 × 10^9^ cells/g, was suspended in DMEM (500 mL), and a T0 count was performed.
A2 An amount of 450 mL of the suspension (step A1) was added to 450 mL of DMEM and 10 g of matrix/100 mL sterile hydroalcoholic solution (90% H_2_0/10% EtOH) (total volume 1 L; matrix 10 G/L).
A3 An amount of 50 mL of the biomass suspension (step A1) + 40 mL DMEM +10 mL sterile hydroalcoholic solution (90% H_2_0/10% EtOH) (total volume 100 mL, the reference preparation was without the matrix).

Cultures A2 (with matrix) and A3 (without matrix) were incubated in anaerobiosis at 37 °C for 72 h.

At the end of the incubation periods (T72), viable counts were performed. Each culture was centrifuged for 20 min at 2,500 RPM, and pH was recorded. pH was adjusted to 7 with 1M NaOH (Merck, Italy). The supernatants were sterilized by filtration through 0.22-μm filters. The resulting solutions were placed in trays, frozen (<−50 °C; >12 h), and lyophilized (>24 h). The powders were packaged in aluminum tri-coupled bags.

##### 2.3.2.2 Process B

B1 An amount of 2 gr of lyophilized *L. paracasei* LMG-S-26420 were inoculated in 500 mL DMEM supplemented with iron, magnesium, manganese, and Tween 80, and a T0 count was performed. The final concentration per L of salts was as follows: FeSO_4_ 0.09 gr; MgSO_4_ 0.3 gr, MnSO_4_ 0.15 gr, and 1 gr of Tween 80 (all reagents were procured from Merck, Italy).
B2 The solution obtained from step B1 was incubated for 24 h at 37 °C in anaerobiosis, and a T24 count was performed.
B3 An amount of 450 mL of the suspension (step B2) was mixed with 450 mL of fresh DMEM +10 g matrix/100 mL sterile hydroalcoholic solution (90% H_2_0/10% EtOH). Culture B3 was left in anaerobiosis at 37 °C for 48 h. At the end of the incubation periods, a CFU count (T72) was performed. The culture was centrifuged for 20 min at 2,500 RPM, and pH was recorded. pH was adjusted to 7 with 1M NaOH. The supernatant was sterilized by filtration through 0.22-μm filters. The resulting solution was frozen (<−50 °C; >12 h) and freeze-dried (>24 h). The powders were packaged in aluminum tri-coupled bags.

##### 2.3.2.3 Process C

Process C was analogous to process B till the pH adjustment to 7. At this level, two different preparations (C1 and C2) were followed. The C1 protocol consisted of the thermal treatment of the fermented product and the biomass at 80 °C for 30′ with the aim of heat-inactivating the bacterial cells. The resulting solution was frozen (<−50 °C; >12 h) and freeze-dried (>24 h). The C2 protocol assessed the separation by centrifugation of the biomass (C2a) from the supernatant (C2b). The first one was treated for 30′ at 80 °C, and the supernatant was frozen at −20 °C. Finally, three products were frozen (<−50 °C; >12 h) and freeze-dried (>24 h). The powders were packaged in aluminum tri-coupled bags.

#### 2.3.3 Viable counts

*L. paracasei* LMG-S-26420 viable counts were performed following the guidelines provided in the document “ISTISAN Reports 2008/36, traditional microbiological methods and molecular methods for the analysis of food supplements based on or with probiotics for human use.” For the *Lactobacillus* genus, this document specifies the use of MRS agar (BD, Difco, Italy) plates inoculated by spreading and incubating for 72 h at 37 °C under anaerobic conditions.

### 2.4 Determination of the anti-inflammatory activity of postbiotics versus matrices

The anti-inflammatory activity of all the matrices investigated (except for M02–M04) toward the corresponding postbiotics, which were obtained by fermentation (process A) with *L. paracasei* LMG-S-26420, was preliminarily investigated. The anti-inflammatory activity of the products under investigation was determined by measuring the levels of an anti-inflammatory cytokine (IL-10) secreted by RAW 264.7 cells after stimulation with lipopolysaccharide (LPS). For this purpose, RAW 264.7 cells, a macrophage cell line obtained from a male mouse tumor induced with Abelson murine leukemia virus, were plated in complete DMEM (Euroclone) supplemented with 10% FBS in 48-well plates at a density of 2 × 10^5^ cells per well. After 24 h, the medium was replaced with fresh, serum-free medium, and each sample in the lyophilic form was added to a well at the concentration of 0.5 mg/mL. An amount of 0.625 mM butyrate (Sigma) was used as an anti-inflammatory positive control. After 3 h of incubation at 37 °C, LPS (derived from *E. coli* 055.B5) (Sigma) was added to each well at a final concentration of 10 μg/mL. Cells were incubated for 18 h at 37 °C. The culture medium was then collected from the wells and centrifuged, and the secreted level of IL-10 was determined by an ELISA using the commercial Mouse IL-10 Uncoated ELISA Kit (Invitrogen, Thermo Fisher Scientific) (Chan et al., 2015).

### 2.5 *In vitro* analysis to evaluate the inhibition of TMA lyase in fecal slurry

The fermentation products obtained were tested to evaluate their ability to inhibit TMA production by the microorganisms present in the human fecal slurry. Subsequently, the experiments were evaluated by measuring the amount of TMA released into the culture medium (Iglesias-Carres et al., 2021). The fecal slurry was prepared from the stool of a healthy volunteer and diluted to a concentration of 100 mg/mL in M9 medium (Sigma-Aldrich, M6030). An amount of 2 mL of fecal slurry was immediately taken in duplicate: one replicate was immediately frozen at −80 °C to have an unstimulated fecal slurry sample for the NGS analysis, whereas the other replicate was acidified with 0.1N HCI (Sigma-Aldrich, 50%/50% v/v), centrifuged at 4 °C at 900 x g for 8 min, and filter-sterilized in a 0.22-μm cellulose acetate filter. The samples obtained were frozen and stored at −80 °C until use for the TMA measurement in the downstream evaluations.

For the inhibition assay, 1,996 μL of fecal slurry was placed in the wells of a deep-well plate, and 2 μL of fermentation products’ solution diluted in sterile water was added at the beginning of the experiment (T0) to obtain a final concentration of 50 μM (or 7.99 × 10^−3^ mg/mL). The samples were incubated for 1 hour at 37 °C in anaerobiosis. After 1 hour (T1), 2 μL of choline chloride (Sigma Aldrich, C7017) (50 M) was added, and the samples were incubated for 3 (T3) or 12 h (T12) from the start of the experiment (T0).

Aliquots at the various time points after fecal slurry inoculation were collected and acidified with 2 mL 0.1N HCl (Sigma-Aldrich, 50%/50% v/v), centrifuged at 4 °C at 900 × g for 8 min, filter-sterilized in a 0.22-μm cellulose acetate filter, and frozen at −80 °C until performing the experiments for the determination of the amount of TMA in the fecal slurry supernatants. The inhibitory activity of the compounds was assessed by determining the concentration of TMA present in the samples. Subsequently, the samples were analyzed by mass spectrometry coupled with LC-MS/MS liquid chromatography.

All fermentation procedures were carried out inside a Ruskinn Concept 400 anaerobic workstation (Baker EMEA, Utrecht, Netherlands). The anaerobic chamber was filled with a mixed gas composed of 5% H_2_, 5% CO_2_, and 90% N_2_ provided by Airgas (Durham, NC, United States). Temperature was set at 37 °C, and it was maintained at a constant value (recorded values were within 36 °C–38 °C) throughout the fermentation procedure.

### 2.6 Chromatographic analysis of TMA-related compounds

TMA and TMAO were measured through LC-MS/MS analysis using a system composed by an UHPLC Ultimate 3000 (Thermo Fisher Scientific) and a Q-Exactive Plus high-resolution mass spectrometer (Thermo Fisher Scientific).

The analyses were performed using a BEH HILIC 1.7 µm 1.0 × 100 mm Acquity UPLC column (Waters). The chromatographic eluent phases were acetonitrile (LC-MS grade, Merck) as phase A and 20 mM ammonium formate in H_2_O (pH 3.5) as phase B. The flow rate was set to 100 μL/min. The chromatographic run was performed in a gradient starting with 90% phase A and 10% phase B and kept constant for 2 min. Successively, phase B was increased up to 50% in 3.5 min and then returned to 10% in 1 min. The column oven temperature was set to 30 °C.

Stable isotope D9-TMA and D9-TMAO (Cambridge Isotope Laboratories, Inc.) were added to the samples as the internal standard for the TMA and TMAO quantification, respectively.

The Q-Exactive Plus Mass Spectrometer (Thermo Fisher Scientific) was equipped with electrospray ionization (ESI) operated in the positive mode: ESI spray voltage +3.8 kV, sheath gas (N_2_) 20 a.u., auxiliary gas (N_2_) 5 a.u., capillary temperature 320 °C, and mass resolution 70,000 at *m/z* 400.

Data were acquired using the PRM mode. For the quantitative determination of TMA, the following transitions were considered: *m/z* 146.1 [M + H]+(of the derivatized TMA) → *m/z* 118.1 as the identifying fragment and *m/z* 155.1 [M + H]+(of the derivatized D9-TMA) → *m/z* 127.1.

TMAO was monitored by the transition *m/z* 76.07 [M + H]+→*m/z* 59.07; meanwhile, for the internal standard D9-TMAO, the transition *m/z* 85.16 for [M + H]+and the fragment ion at *m/z* 69.14 were considered.

### 2.7 TMA-related compounds’ sample quantification

The biological sample (slurry, urine, and plasma) preparation for TMA quantification is briefly described below. An amount of 25 μL of each starting sample was collected after vortexing for 5 min. An amount of 20 μL of D9-trimethylamine-DCl, 8 µL of ammonia, and 120 µL of ethyl bromoacetate (20 mg/mL), all procured from Merck, were added. The mixture was allowed to react for 30 min. Then, 120 µL of a 0.025% (v/v) solution of formic acid in H_2_O/acetonitrile (50/50, v/v) was added. The resulting mixture was centrifuged, the supernatant was removed, and the sample was subjected to the LC-MS/MS analysis without further manipulation. The derivatization protocol for the quantification of TMA was an adaptation of the protocol described by Iglesias-Carres et al. (2021).

Samples for TMAO levels’ quantification were prepared as follows: an amount of 25 μL of each sample was collected, and 3 μL of D9-TMAO (5 μM concentration) and 122 μL of a cold acetonitrile:methanol mixture (50:50, v/v) were added for protein precipitation. After centrifuging at 13,000 rpm for 10 min at 4 °C, the supernatant was removed and dried with nitrogen. The residue was resuspended with 25 μL of a solution consisting of 90% acetonitrile and 10% 20 mM ammonium formate at pH 3.5 (v/v). The samples were diluted 60 times (final volume of 300 μL). An amount of 10 μL was used for the LC-MS/MS analysis described above.

The TMA and TMAO calibration curves were obtained by adding a known amount of D9-TMA or D9-TMAO to all dilutions of the TMA or TMAO standards, respectively. Samples related to the calibration curve were processed in the same manner adopted for the biological samples. An amount of 10 μL of each standard mixture, containing the labeled internal standard, was used for the LC-MS/MS analysis. The areas thus obtained for standard TMA were correlated with the area of the internal standard, and the resulting ratio (TMA/D9-TMA or TMAO/D9-TMAO) was correlated with the standard concentration to verify the range of linearity of the response, which was in the range from 25 nM to 100 μM. In order to validate the analytical methods, the standard curve was acquired in triplicate and in three different days. The resulting curve was used to determine the concentration of TMA in each of the samples under analysis.

### 2.8 Cell count

The number of cells present in the fermentation media was approximated by reading the optical density at 600 nm in a SpectraMax iD3 plate reader (Molecular Devices, San Jose, CA, United State). Cell count was performed in 100_L of the sample drawn at the last time point of fermentation (12 h–36 h), which depended on the experimental scheme.

### 2.9 Animal studies

All experimental procedures were approved by the organization responsible for animal welfare and by the Ethics Committee of the University of Messina (protocol no. 0082350).

A total of 48 female *Mus musculus* C57BL/6 (FMO3 +/+) strain WT and 40 female *Mus musculus* C57BL/6-Fmo3^em1Smoc^ (KO) mice (Cat. No. NM-KO-200734) were purchased, respectively, from Charles Rivers laboratories and Shanghai Model Organisms Center. All animals, which were 8 weeks old, were housed in the “G. Barresi” Department of Human Pathology of the adult and developmental-age animal house, the University of Messina. Both wild-type and knockout mice were divided into eight and four groups, respectively, and maintained under a 12:12 h light–dark cycle in irradiated poplar litter. During the experimentation, the following clinical parameters were monitored daily: coat status, kyphosis, reduced mobility, weight loss, and body temperature. All nutrients were irradiated to avoid pathogen contamination. Choline chloride (AC81182) and fluorocholine chloride (AI50690) were purchased from A2B Chem LLC (Saint Diego, California). Choline bitartrate (0.08%) was purchased from Mucedola S.R.L. (Milan).

#### 2.9.1 Phase 1

A total of 48 female *Mus musculus* C57BL/6 (FMO3 +/+) strain WT mice, who were 8 weeks old, were divided into eight groups: six groups were based on the compound to be tested and the other two groups were, respectively, the positive control (FMC treatment) and the negative control (only phosphate-buffered saline treatment). Each compound, diluted in 0.2 mL of phosphate-buffered saline (PBS), was administered via oral gavage, as shown in Supplementary Table 2. The choline chloride (AC81182) load was administered by oral gavage at 2 mg dosage per mouse. The experimental protocol was performed 4 days after previous acclimatization. On the first day, all mice groups were fed a low-choline diet (choline bitartrate 0.08%), and fecal samples from each experimental group were collected and stored at −80 °C for subsequent gut microbiota typing analysis. On the second day, for all the groups, the different compounds were administered via oral gavage after 1-hour fasting, followed by the choline chloride load the next hour. At 3 h after treatment, 50 µL of blood was taken from the submandibular vein and centrifuged at 4 °C and 4,000 rpm for 20 min to separate and collect plasma. At 6 h after treatment, fecal samples were collected. Both samples were stored at −80 °C. The same protocol was performed on days 3 and 4, but on day 3, no sample was collected (Roberts et al., 2018; Pathak et al., 2020).

#### 2.9.2 Phase 2

The second phase of the experiment was conducted on 8-week-old female C57BL/6-Fmo3^em1Smoc^ (KO) mice, in which exons 3–6 of the *FMO3* gene were deleted to generate *FMO3* knockout mice. The composition of the intestinal microbiota was characterized, following its removal and subsequent fecal transplant from a human donor (Bokoliya et al., 2021). The latter, who was “appropriately” informed, consented to the use of their stool sample for research purposes. The two compounds to be tested were administered by gavage according to the attached protocol. TMA and TMAO levels were determined in urine and blood samples. A total of 40 *Mus musculus* C57BL/6 Fmo3^em1Smoc^ (KO) mice were divided into four groups, and for each group, two replicates were performed, as indicated in Supplementary Table 3. All 40 female *Mus musculus* C57BL/6-Fmo3^em1Smoc^ (KO) mice were treated for 12 days with a mix of selected antibiotics—amoxicillin (Sigma-Aldrich, cat. 26787-78-0) + neomycin (Sigma-Aldrich, cat. N1142) + metronidazole (Sigma-Aldrich, cat. 443-48-1) + vancomycin (Millipore, cat. 1404-93-9)—in the ratio 1:1:1:0.5 g/dL (10 mg/mouse). Subsequently, they underwent six cycles of fecal transplants that were evenly distributed over 21 days, thus numbering to three a week, respectively.

After completing this step, the experimental protocol was performed in 9 days. On the first day, all mice groups were fed a low-choline diet (choline bitartrate 0.08%), and fecal samples from each experimental group were collected and stored at −80 °C for subsequent gut microbiota typing analysis. Furthermore, 50 µL of blood was taken from the submandibular vein and centrifuged at 4 °C and 4,000 rpm for 20 min to collect plasma; urine samples were also collected.

On the second day, all animal groups were administered different compounds via oral gavage after 1 hour of fasting, followed, the next hour, by the choline chloride load. In 3 h after treatment with the compounds, a blood sample was taken from each mouse, and 6 h after treatment, urine samples were collected. Both samples were stored at −80 ° C for subsequent analysis.

From the third day to the seventh day, all mice were fed with a normal diet. Test compounds were administered via oral gavage to all mice groups after 1 hour of fasting.

On the eighth day, no treatment was performed, but all animal groups were fed a low-choline diet (choline bitartrate 0.08%), and blood and urine samples were collected. Finally, on the ninth day, the same protocol was performed as on the second day. All samples were stored at −80 ° C for subsequent analysis. Moreover, all the animals were sacrificed at the end of the experiment.

### 2.10 Gut microbiota analysis

#### 2.10.1 NGS amplicon sequencing

The microbiota in the fecal slurry was analyzed using the NGS technology. The DNA was extracted and purified from the sample using the DNeasy PowerSoil Pro Kit (Qiagen Italia, Milan, Italy). The fragments to be sequenced were produced following the Illumina kit protocol, amplifying the V3–V4 region of the 16S rRNA gene. The fragment pools produced (libraries) were diluted to the concentration of 4 nM, denatured, and diluted to 5 pM. A total of 30% of equimolar PhiX was added, and the reaction was loaded onto the MiSeq platform to be sequenced with the MiSeq V2 2 × 250 bp Illumina kit [Illumina, Inc.; cat. MS-102-3003]. To eliminate the risk of evaluating contaminating microorganisms from the environment in downstream analysis, negative (sample-free) controls were processed simultaneously with the samples of the experiment.

#### 2.10.2 Data analysis and data processing

The sequences obtained were analyzed using the bioinformatics platform QIIME2™ version 2020.6. The DADA2 (divisive amplicon denoising algorithm 2) algorithm (Hall M., 2018) was used to remove background noise and chimeras and generate ASVs (amplicon sequence variants). Quality filtering and clustering were performed with the open-source tool VSEARCH 2020.6.0 (Rognes et al., 2016). Taxonomic classification was performed on the SILVA database version 132. The downstream analysis was carried out using the free software environment R version 4.0.3 and the open-source integrated development environment (IDE) RStudio (https://posit.co/products/open-source/rstudio/) v1.4.1103, and the environmental contaminants were excluded using the open-source *decontam* R package (https://github.com/benjjneb/decontam) (Davis et al., 2018). Sequencing data of the fecal slurries were normalized by rarefaction without replacement in RStudio prior downstream analysis. Alpha diversity differences between the groups were evaluated using ANOVA and Tukey’s HSD (honestly significant difference) tests for normally distributed data or the Wilcoxon–Mann–Whitney test with the Holm–Bonferroni correction method (WMW with HB) for non-normally distributed data. Using the R *phyloseq* package (McMurdie and Holmes, 2013), the relative abundance data (RA%) of the bacterial taxa present in the slurry were obtained up to the genus taxonomic level. The permutational analysis of variance (PERMANOVA) test, calculated using the function *adonis2* in the *vegan* R package, was performed to determine whether there was a significant separation between different sample groups (https://cran.r-project.org/web/packages/vegan/vegan.pdf, accessed on 22 December 2023). The linear discriminant analysis (LDA) effect size (LEfSe) algorithm, a tool which is hosted on the Galaxy web application at https://huttenhower.sph.harvard.edu/galaxy/(accessed on 22 December 2023), was used to discover bacterial taxa associated with the treatment (Segata et al., 2011).

## 3 Results

### 3.1 Pilot scale fermentation of *L. paracasei* LMG-S-26420

**FIGURE 2:**
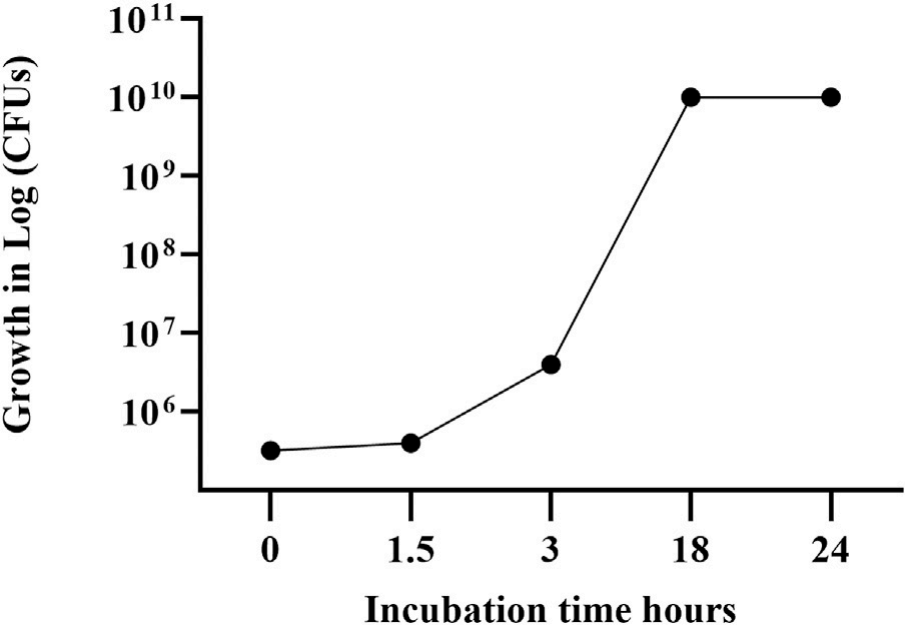
Growth curve of strain LMG S-26420 in MRS medium during bioreactor fermentation. The data shown represent the mean of two independent experiments, with standard deviations below 6% (range: 2%–6%).

Figure 2 reports the growth curve data separately for the sampling time points during the bioreactor growth steps of the *L. paracasei* LMG-S-26420 strain.

The fresh biomass of the *L. paracasei* LMG S-26420 strain was resuspended with maltodextrin solution, obtaining a suspension carrying 2 × 10^11^ viable cells per ml. Following lyophilization, the powder contained 4.1 × 10^12^ CFUs per gram.

Two grams of *L. paracasei* LMG-S-26420 freeze-dried powder were used for processes B and C for each tested matrix. The quantities of freeze-dried powders obtained are indicated in Table 1.

**TABLE 1:** Quantity of lyophilized powders obtained during the production assay.

Product	gr
Reference preparation Culture A3 (without matrix) AAT-01/Ref	3.5
AL0010 AAT-02/M05/A	3.7
AL0010 AAT-03/M05/B	4.9
AL0044 AAT-04/M05/C1	4
AL0044 AAT-05/M05/C2a	0.29
AL0044 AAT-06/M05/C2b	5
AL0011 AAT-07/M06/A	5
AL0011 AAT-08/M06/B	4.1
AL0045 AAT-09/M06/C1	3.6
AL0045 AAT-10/M06/C2a	0.35
AL0045 AAT-11/M06/C2b	4
AL0012 AAT-12/M09/A	3.3
AL0012 AAT-13/M09/B	4.4
AL0046 AAT-14/M09/C1	4.9
AL0046 AAT-15/M09/C2a	2.1
AL0046 AAT-16/M09/C2b	4.4
AL0013 AAT-17/M11/A	6.7
AL0013 AAT-18/M11/B	5.4
AL0047 AAT-19/M11/C1	5.6
AL0047 AAT-20/M11/C2a	0.8
AL0047 AAT-21/M11/C2b	5.15

### 3.2 Growth results in the tested matrices

**FIGURE 3:**
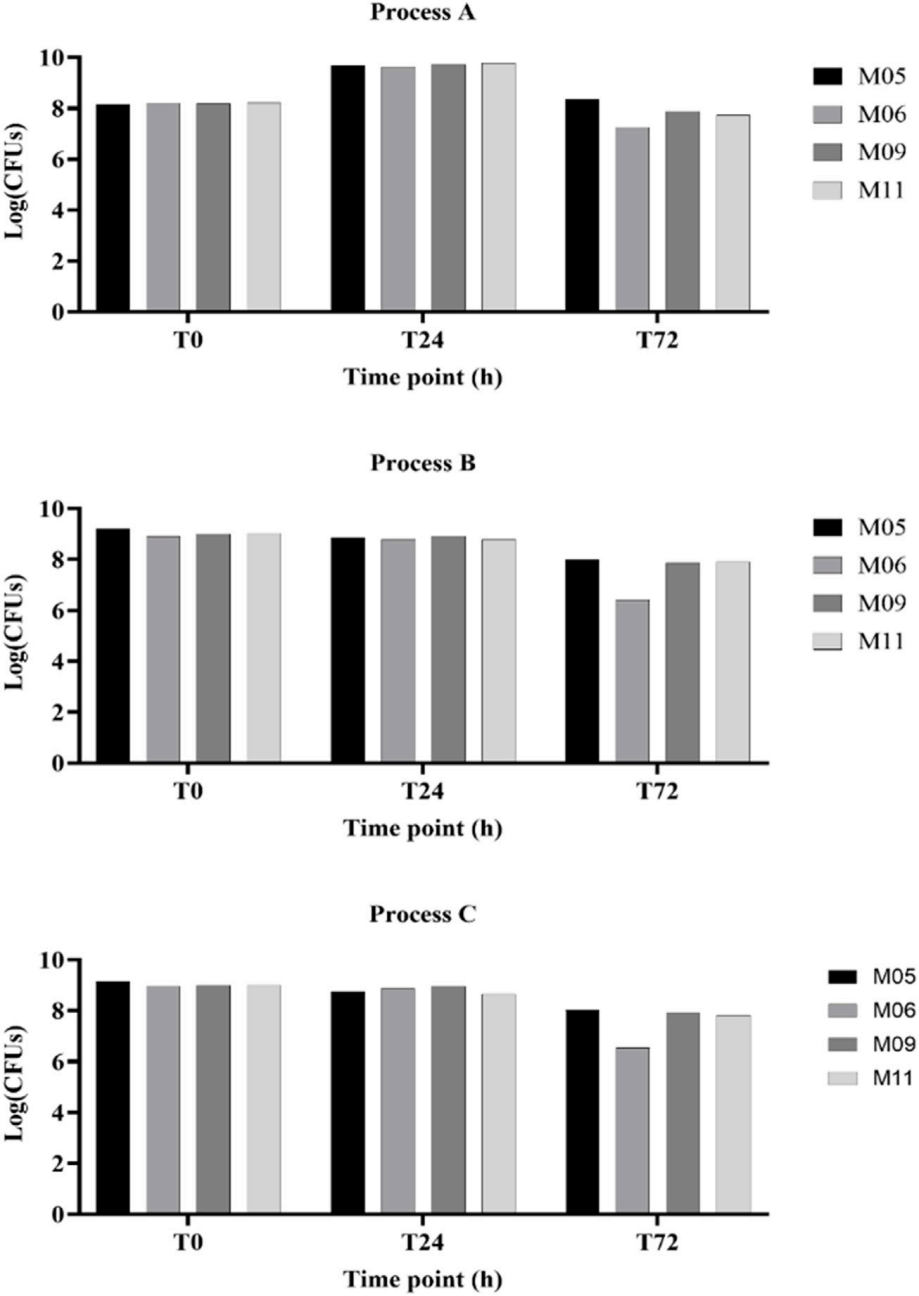
Yields of viable cell concentrations obtained with the four matrices (MI 05, MI 06, MI 09, and MI 11) following the different fermentation processes (indicated as A, B, and C).

Table 2 and Figure 3 collectively illustrate the growth dynamics of the *L. paracasei* LMG S-26420 strain in four different matrices (M05, M06, M09, and M11) under three fermentation processes (A, B, and C). During the first 24 h, an increase in viable counts was observed under process A across all matrices (DeltaLog (T24–T0), ranging from 1.43 to 1.56), indicating favorable growth conditions. In contrast, processes B and C resulted in limited or negative changes in cell counts. From 24 to 72 h, a general decline in viability was observed under all conditions, with the most pronounced reductions occurring under process A in matrices M06 and M11 (DeltaLog (T72–T24) of −2.38 and −2.04, respectively). Among the tested matrices, M05 and M09 demonstrated better retention of viable cells during the later fermentation phase, as supported by both DeltaLog (CFUs) values’ trends.

**TABLE 2:** DeltaLog transformed growth data (Log (CFUs/g)) for *L. paracasei* LMG-S-26420 strain for the analyzed matrices (M05, M06, M09, and M11) considering the three different processes (indicated as A, B, and C1) and the time points (T0, T24, and T72).

Process	DeltaLog (T24–T0)	DeltaLog (T72–T24)
M05		
A	1.54	−1.33
B	−0.35	−0.86
C1	−0.40	−0.71
M06		
A	1.43	−2.38
B	−0.15	−2.35
C1	−0.10	−2.33
M09		
A	1.56	−1.85
B	−0.06	−1.09
C1	−0.03	−1.07
M11		
A	1.55	−2.04
B	−0.26	−0.85
C1	−0.35	−0.83

### 3.3 Results of the anti-inflammatory activity

Typically, postbiotics were more endowed with anti-inflammatory activity than the corresponding matrices, as shown by the test results. The secreted IL-10 cytokine values measured in the supernatant collected from cells stimulated with LPS and treated with the test products are reported in Table 3, which are expressed in terms of “percentage” (%) with respect to the cytokine level measured in the supernatant collected from control cells, stimulated with LPS, and incubated with the medium alone. In the supernatant alone, where *L. Paracasei* was left to ferment for 72 h without the matrix, IL-10 secretion increased by 180% compared with the control. Based on other preliminary tests, postbiotics obtained from the fermentation of *L. paracasei* with matrices M05–M06–M09–M11, AL0044–AL0045–AL0046–AL0047, respectively, were candidates for this study. Notably, AL0044, AL0045, AL0046, and AL0047 are “postbiotics + tyndallized probiotics,” whereas AL0010, AL0011, AL0012, and AL0013 are the corresponding “postbiotics alone,” without the probiotic component.

**TABLE 3:** IL-10 cytokine values measured in the supernatant collected from cells stimulated with LPS following treatment with postbiotics or the related matrices alone.

Matrix (freeze-dried extract)	Postbiotic 72 h (*L. paracasei* LMG-S-26420)	IL-10 (%) vs*.* ctr
M01, *Curcuma longa*		100^
	Post72/01/Lp	100^
M2, green tea (*Camellia sinensis*)		N.D.*
	Post72/02/Lp	N.D.*
M3, *Ginkgo biloba*		N.D.**
	Post72/03/Lp	N.D.**
M4, grape seed (*Vitis vinifera*)		N.D.***
	Post72/04/Lp	N.D.***
M05, *Allium sativum*		100^
	Post72/05/Lp	**4060**
M06, *Salvia miltiorrhiza*		100^
	Post72/06/Lp	100^
M07, *Zingiber officinale*		100^
	Post72/07/Lp	300
M08, *Myrciaria dubia*		100^
	Post72/08/Lp	**3360**
M09, *Garcinia cambogia*		100^
	Post72/09/Lp	**3570**
M10, *Garcinia mangostana*		100^
	Post72/10/Lp	**2510**
M11, *Cassia angustifolia* leaves		310
	Post72/11/Lp	340
M12, *Nelumbo nucifera* leaves		280
	Post72/12/Lp	100^
M13, *Boswellia carterii serrata*		330
	Post72/13/Lp	**3490**
M14, broccoli sprouts		100^
	Post72/14/Lp	**2610**
M15, *Scutellaria baicalensis Georgi*		100^
	Post72/15/Lp	190
M16, green coffee		100^
	Post72/16/Lp	100^
M17, *Phaseolus vulgaris*		320
	Post72/17/Lp	**3550**
M18, *Phaseolus radiatus*		100^
	Post72/18/Lp	100^
M19, *Lippia citriodora*		100^
	Post72/19/Lp	**2340**
M20, *Hibiscus sabdariffa*		100^
	Post72/20/Lp	**1960**
M21, thorn date seed (*Ziziphus jujuba Mill. var. Spinosa*)		130
	Post72/21/Lp	100
	Supernatant without matrix	180

Cytokine levels are expressed as percentage with respect to those measured in control cells (whose IL-10 level is set at 100%). 100^: no significant changes compared to control cells. N.D. = not determined. * very poor solubility in culture medium (drug precipitation) and toxicity against cells. ** poor solubility in culture medium. *** poor solubility in culture medium and toxicity against cells. Bold values in the “IL-10 (%) vs ctr” column highlight the cytokine levels increased more than 10-fold (> 1000%) with respect to ctr cells.

### 3.4 Results of the *in vitro* TMA lyase inactivation test

The fecal slurries were tested against all the lyophilized powders obtained during the production assays, and at 3 h after the beginning of the experiments, the supernatants were collected to assess the TMA-inhibitory activity. The first column of Table 4 shows the identification of the analyzed samples, and the second column shows the TMA levels measured and expressed in mg/L. The fecal slurry used for all the tested conditions was analyzed by NGS to assess the presence of bacterial TMA producers. The NGS analysis showed that the most represented bacterial taxa capable of producing TMA in the fecal slurry samples were the genera *Collinsella*, *Clostridium*, and *Streptococcus*. The relative abundances results (RA%) are shown in Table 5 along with the complete taxonomic information of the bacterial taxa found in the fecal slurry from the Kingdom to the Genus level.

**TABLE 4:** TMA levels measured in fecal slurries tested against all the lyophilized powders.

Samples	TMA levels (mg/L)
Slurry T0 (ctrl)	2.8 ± 0.4
Slurry + AAT-01/Ref + COL 50	19.5 ± 1.0
Slurry + AL0010 (AAT-02/M05/A) + COL 50	7.9 ± 0.7
Slurry + AL0010 (AAT-03/M05/B) + COL 50	7.8 ± 0.5
Slurry + AL0044 (AAT-04/M05/C1) + COL 50	8.6 ± 1.2
Slurry + AL0044 (AAT-05/M05/C2a) + COL 50	16.6 ± 2.2
Slurry + AL0044 (AAT-06/M05/C2b) + COL 50	8.3 ± 0.3
Slurry + AL0011 (AAT-07/M06/A) + COL 50	11.6 ± 1.1
Slurry + AL0011 (AAT-08/M06/B) + COL 50	6.8 ± 0.9
Slurry + AL0045 (AAT-09/M06/C1) + COL 50	23.1 ± 2.1
Slurry + AL0045 (AAT-10/M06/C2a) + COL 50	16.6 ± 3.0
Slurry + AL0045 (AAT-11/M06/C2b) + COL 50	20.9 ± 2.5
Slurry + AL0012 (AAT-12/M09/A) + COL 50	14.1 ± 3.2
Slurry + AL0012 (AAT-13/M09/B) + COL 50	18.0 ± 3.0
Slurry + AL0046 (AAT-14/M09/C1) + COL 50	13.3 ± 2.8
Slurry + AL0046 (AAT-15/M09/C2a) + COL 50	13.2 ± 0.4
Slurry + AL0046 (AAT-16/M09/C2b) + COL 50	9.5 ± 2.1
Slurry + AL0013 (AAT-17/M11/A) + COL 50	10.3 ± 3.6
Slurry + AL0013 (AAT-18/M11/B) + COL 50	19.8 ± 4.7
Slurry + AL0047 (AAT-19/M11/C1) + COL 50	7.0 ± 4.9
Slurry + AL0047 (AAT-20/M11/C2a) + COL 50	9.2 ± 2.5
Slurry + AL0047 (AAT-21/M11/C2b) + COL 50	5.4 ± 2.7
Slurry + COL 50 (ctrl)	5.4 ± 2.9
Slurry T3 (ctrl)	11.4 ± 3.5

**TABLE 5:** Relative abundance data (RA%) of the bacterial genera present in the fecal slurry.

Bacterial genus	RA (%) in the fecal slurry
*Bacteria; Bacteroidetes; Bacteroidia; Bacteroidales; Prevotellaceae; Prevotella 9*	18.75
*Bacteria; Firmicutes; Clostridia; Clostridiales; Ruminococcaceae; Faecalibacterium*	13.17
*Bacteria; Firmicutes; Clostridia; Clostridiales; Lachnospiraceae; Lachnospiraceae UCG-008*	5.36
*Bacteria; Bacteroidetes; Bacteroidia; Bacteroidales; Bacteroidaceae; Bacteroides*	4.26
*Bacteria; Actinobacteria; Actinobacteria; Bifidobacteriales; Bifidobacteriaceae; Bifidobacterium*	3.81
*Bacteria; Firmicutes; Clostridia; Clostridiales; Lachnospiraceae; Fusicatenibacter*	3.74
*Bacteria;Firmicutes; Clostridia; Clostridiales; Ruminococcaceae; Subdoligranulum*	3.65
*Bacteria; Firmicutes; Clostridia; Clostridiales; Ruminococcaceae; Ruminococcus 2*	3.33
*Bacteria; Firmicutes; Clostridia; Clostridiales; Lachnospiraceae; Roseburia*	2.82
*Bacteria; Firmicutes; Negativicutes; Selenomonadales; Veillonellaceae; Mitsuokella*	2.56
*Bacteria; Firmicutes; Clostridia; Clostridiales; Lachnospiraceae; Anaerostipes*	2.37
*Bacteria; Firmicutes; Clostridia; Clostridiales; Lachnospiraceae*	2.29
*Bacteria; Firmicutes; Clostridia; Clostridiales; Lachnospiraceae; Lachnospira*	2.16
*Bacteria; Firmicutes; Clostridia; Clostridiales; Lachnospiraceae; [Eubacterium] hallii group*	2.13
*Bacteria; Firmicutes; Clostridia; Clostridiales; Lachnospiraceae; Blautia*	2.12
*Bacteria; Firmicutes; Clostridia; Clostridiales; Lachnospiraceae; [Eubacterium] ruminantium group*	1.99
***Bacteria; Actinobacteria; Coriobacteriia; Coriobacteriales; Coriobacteriaceae; Collinsella***	1.89
*Bacteria; Bacteroidetes; Bacteroidia; Bacteroidales; Tannerellaceae; Parabacteroides*	1.84
***Bacteria; Firmicutes; Bacilli; Lactobacillales; Streptococcaceae; Streptococcus***	1.45
*Bacteria; Firmicutes; Clostridia; Clostridiales; Ruminococcaceae; Ruminococcus 1*	1.38
*Bacteria; Firmicutes; Clostridia; Clostridiales; Peptostreptococcaceae; Romboutsia*	1.35
*Bacteria; Firmicutes; Erysipelotrichia; Erysipelotrichales; Erysipelotrichaceae; Holdemanella*	1.33
*Bacteria; Firmicutes; Negativicutes; Selenomonadales; Veillonellaceae; Dialister*	1.33
*Bacteria; Firmicutes; Negativicutes; Selenomonadales; Acidaminococcaceae; Phascolarctobacterium*	1.31
*Bacteria; Firmicutes; Clostridia; Clostridiales; Ruminococcaceae; [Eubacterium] coprostanoligenes group*	1.23
*Bacteria; Firmicutes; Clostridia; Clostridiales; Lachnospiraceae; Dorea*	1.06
*Bacteria; Firmicutes; Clostridia; Clostridiales; Lachnospiraceae; Coprococcus 3*	0.88
*Bacteria; Firmicutes; Clostridia; Clostridiales; Lachnospiraceae; Lachnospiraceae ND3007 group*	0.84
*Bacteria; Firmicutes; Clostridia; Clostridiales; Lachnospiraceae; [Eubacterium] eligens group*	0.76
*Bacteria; Firmicutes; Clostridia; Clostridiales; Ruminococcaceae; Ruminococcaceae NK4A214 group*	0.64
*Bacteria; Actinobacteria; Coriobacteriia; Coriobacteriales; Atopobiaceae; Coriobacteriaceae UCG-003*	0.63
*Bacteria; Firmicutes; Negativicutes; Selenomonadales; Acidaminococcaceae; Acidaminococcus*	0.63
*Bacteria; Proteobacteria; Gammaproteobacteria; Betaproteobacteriales; Burkholderiaceae; Sutterella*	0.58
*Bacteria; Actinobacteria; Coriobacteriia; Coriobacteriales; Atopobiaceae; Libanicoccus*	0.51
*Bacteria; Firmicutes; Clostridia; Clostridiales; Lachnospiraceae; Lachnospiraceae NK4A136 group*	0.51
*Bacteria; Firmicutes; Clostridia; Clostridiales; Lachnospiraceae; Lachnospiraceae FCS020 group*	0.42
*Bacteria; Firmicutes; Clostridia; Clostridiales; Lachnospiraceae; Lachnospiraceae UCG-004*	0.38
*Bacteria; Verrucomicrobia; Verrucomicrobiae; Verrucomicrobiales; Akkermansiaceae; Akkermansia*	0.37
*Bacteria; Firmicutes; Clostridia; Clostridiales; Ruminococcaceae; Ruminococcaceae UCG-002*	0.34
*Bacteria; Actinobacteria; Coriobacteriia; Coriobacteriales; Eggerthellaceae; Senegalimassilia*	0.32
*Bacteria; Firmicutes; Clostridia; Clostridiales; Lachnospiraceae; Coprococcus 2*	0.32
*Bacteria; Firmicutes; Clostridia; Clostridiales; Lachnospiraceae; Lachnospiraceae UCG-001*	0.31
*Bacteria; Firmicutes; Clostridia; Clostridiales; Lachnospiraceae; Lachnoclostridium*	0.27
*Bacteria; Firmicutes; Clostridia; Clostridiales; Ruminococcaceae; Butyricicoccus*	0.23
*Bacteria; Actinobacteria; Coriobacteriia; Coriobacteriales; Eggerthellaceae; Slackia*	0.23
*Bacteria; Bacteroidetes; Bacteroidia; Bacteroidales; Prevotellaceae; Paraprevotella*	0.23
*Bacteria; Firmicutes; Clostridia; Clostridiales; Ruminococcaceae; Ruminococcaceae UCG-004*	0.19
*Bacteria; Firmicutes; Clostridia; Clostridiales; Ruminococcaceae; Ruminococcaceae UCG-003*	0.18
*Bacteria; Firmicutes; Clostridia; Clostridiales; Lachnospiraceae; uncultured*	0.17
*Bacteria;Firmicutes; Clostridia; Clostridiales; Ruminococcaceae; Ruminiclostridium 9*	0.16
*Bacteria; Firmicutes; Erysipelotrichia; Erysipelotrichales; Erysipelotrichaceae; Turicibacter*	0.15
***Bacteria; Firmicutes; Clostridia; Clostridiales; Clostridiaceae 1; Clostridium sensu stricto 1***	0.14
*Bacteria; Firmicutes; Erysipelotrichia; Erysipelotrichales; Erysipelotrichaceae; Erysipelotrichaceae UCG-003*	0.12
*Bacteria;Firmicutes;Clostridia;Clostridiales;Ruminococcaceae;Ruminococcaceae UCG-005*	0.10
*Bacteria;Bacteroidetes;Bacteroidia;Bacteroidales;Marinifilaceae;Butyricimonas*	0.09
*Bacteria; Tenericutes; Mollicutes; Mollicutes RF39; uncultured bacterium; uncultured bacterium*	0.09
*Bacteria; Firmicutes; Clostridia; Clostridiales; Christensenellaceae; Christensenellaceae R-7 group*	0.08
*Bacteria; Firmicutes; Clostridia; Clostridiales; Ruminococcaceae; Ruminiclostridium 5*	0.08
*Bacteria; Actinobacteria; Coriobacteriia; Coriobacteriales; Eggerthellaceae; Eggerthella*	0.07
*Bacteria; Firmicutes; Clostridia; Clostridiales; Lachnospiraceae; [Eubacterium] xylanophilum group*	0.06
*Bacteria; Firmicutes; Clostridia; Clostridiales; Family XIII; Family XIII AD3011 group*	0.05
*Bacteria; Firmicutes; Erysipelotrichia; Erysipelotrichales; Erysipelotrichaceae; Erysipelatoclostridium*	0.04
*Bacteria; Firmicutes; Clostridia; Clostridiales; Ruminococcaceae; Ruminococcaceae UCG-014*	0.03
*Bacteria; Firmicutes; Clostridia; Clostridiales; Ruminococcaceae; uncultured*	0.03
*Bacteria; Firmicutes; Clostridia; Clostridiales; Ruminococcaceae; Anaerofilum*	0.02
*Bacteria; Firmicutes; Clostridia; Clostridiales; Ruminococcaceae*	0.02
*Bacteria; Firmicutes; Clostridia; Clostridiales; Lachnospiraceae; Marvinbryantia*	0.01
*Bacteria; Firmicutes; Clostridia; Clostridiales; Lachnospiraceae; [Ruminococcus] torques group*	0.01
*Bacteria;Firmicutes;Clostridia;Clostridiales;Ruminococcaceae;Oscillibacter*	0.01
*Bacteria; Firmicutes; Clostridia; Clostridiales; Lachnospiraceae; [Ruminococcus] gauvreauii group*	0.01

The genera in bold are the bacterial TMA producers.

### 3.5 Determination of plasma TMAO levels in C57Bl/6J mice

The TMAO plasma levels in wild-type mice were determined by LC-MS/MS, as described in the experimental section. The results obtained are summarized in Table 6. The first column reports the experimental group. The second column provides further specification within each group of plant extract. The third column lists the TMAO measured concentrations, expressed in mg/L. The last column shows the calculated average percentage of inhibition. The reported data referred to values detected in the blood samples of mice treated with the product described in column two of Table 6 at day 3, 2 h after choline administration. These data confirmed the inhibition activity of the selected products.

**TABLE 6:** TMAO value in blood samples from wild-type mice after 3 days of treatment.

Product	Matrix	Process	TMAO (mg/L)	% Inhibition (average)
*Allium sativum*	*Allium sativum*		*4.5*	19
*AL0044* AAT-04/M05-C1	*Allium sativum*	*C*	*1.6*	71
*AL0010* AAT-02/M05/A	*Allium sativum*	*A*	*2.0*	57
Leaves of *Cassia angustifolia*	Leaves of *Cassia angustifolia*		*5.6*	*-*
*AL0047* AAT-19/M11/C1	Leaves of *Cassia angustifolia*	*C*	*2.0*	64
*AL0013* AAT-17/M11/A	Leaves of *Cassia angustifolia*	*A*	*2.5*	55
*FMC* (*positive control*)			*0.05*	>95
*PBS* (*negative control*)			*5.5*	*-*

### 3.6 Determination of urine TMA levels in C57Bl/6-Fmo3^em1smoc^ mice

TMA levels were evaluated in urine collected from C57Bl/6-Fmo3^em1smoc^ mice, which lack the Fmo3 enzyme responsible for the conversion of TMA to TMAO. In addition, in this case, the results are summarized in Table 7. The first and second columns of the table report a description of the experimental group and the products used for the treatment. The third column is divided into three sections, and the TMA values measured for urine samples after 1 (D1), 2 (D2), 3 (D3), and 4 (D4) days, expressed in mg/L, are listed.

**TABLE 7:** TMA value in urine of C57Bl/6-Fmo3^em1smoc^ mice.

Product	TMA (mg/L)				% Inhibition (average)
	DAY 1	DAY 2	DAY 3	DAY 4	
AL0044 AAT-04/M05-C1	41.2 ± 11.0	92.0 ± 33.0	23.9 ± 2.0	26.8 ± 13.0	67
AL0047 AAT-19/M11/C1	41.1 ± 24.0	94.7 ± 49.0	79.1 ± 29.0	43.6 ± 11.0	54
FMC	105.5 ± 7.0	16.4 ± 6.0	15.1 ± 4.0	2.8 ± 1.0	75
PBS	119.0 ± 9.0	149.5 ± 20.0	138.5 ± 14.0	146.9 ± 23.0	-

The columns (DAY 1, DAY 2, DAY 3, and DAY 4) represent the average values with the standard deviations.

The inhibitory effect of the fermentation product was evident from the measured TMA values, and the percentage of inhibition was calculated (see column 4 in Table 7 reported above).

### 3.7 Determination of plasma TMA levels in C57Bl/6-Fmo3^em1smoc^ mice

Using the same method as for urine samples, TMA levels were also measured in plasma samples collected from the same experiment performed on C57Bl/6-Fmo3^em1smoc^ mice.

The TMA plasma concentration was evaluated at the same time point chosen for the urine samples, and the obtained values are listed in Table 8.

**TABLE 8:** TMA value in blood of C57Bl/6-Fmo3^em1smoc^ mice.

Product	TMA (mg/L)				% Inhibition (average)
	DAY 1	DAY 2	DAY 3	DAY 4	
AL0044 AAT-04/M05-C1	10.0 ± 1.0	630.1 ± 60.0	230.9 ± 60.0	120.2 ± 40.0	81
AL0047 AAT-19/M11/C1	40.1 ± 1.0	1410.2 ± 220.0	8000.3 ± 420.0	420.1 ± 200.0	49
FMC	40.1 ± 1.0	140.2 ± 30.0	250.3 ± 20.0	90.4 ± 10.0	91
PBS	170.0 ± 9.0	2090.5 ± 590.0	1040.5 ± 100.0	1920.1 ± 690.0	-

The columns (DAY 1, DAY 2, DAY 3, and DAY 4) represent the average values with the standard deviations.

In addition, in this case, the TMA levels were reduced after treatment with fermented products, and the measured values were nearly comparable to those obtained from treatment with FMC, a TMA lyase inhibitor considered as the reference compound.

### 3.8 Results deriving from bacterial microbiota analysis in C57Bl/6-Fmo3^em1smoc^ mice

**FIGURE 4:**
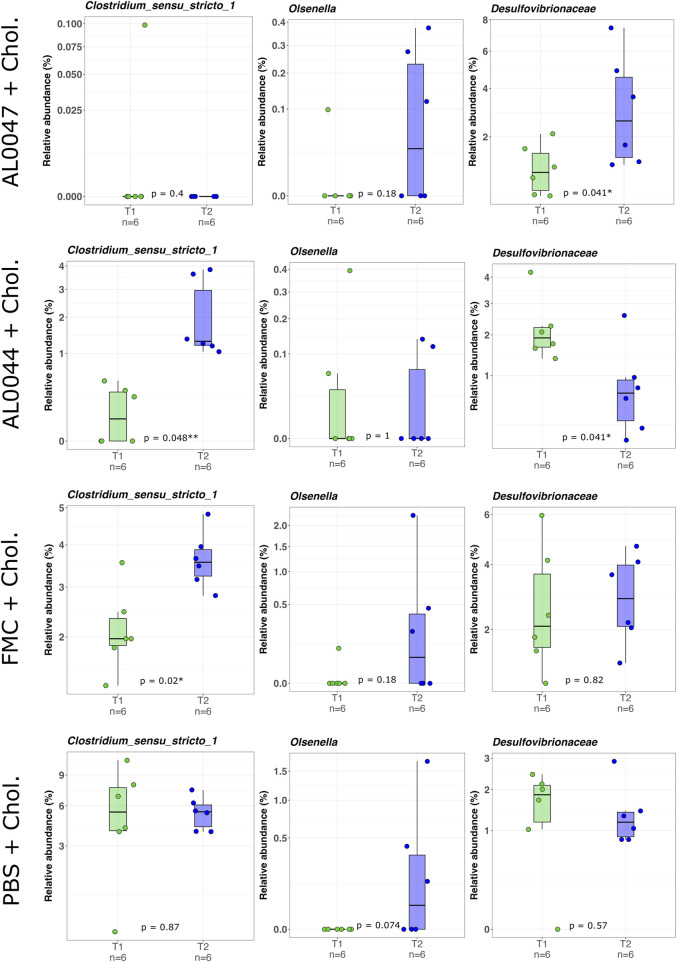
Box-and-whisker plots with data points showing the relative abundance percentage (RA%) of bacterial TMA producers present in the mouse intestine before (T1) and after (T2) the treatments. The first two rows show the genera of TMA producers (*Clostridium sensu stricto* (Sorboni et al., 2022) and *Olsenella*), whereas the third one shows a family of known TMA-producers (Desulfovibrionaceae). Mann–Whitney U test result of the group comparison is shown in each panel. * FDR-corrected *p* < 0.05; ** FDR-corrected *p* < 0.01. FMC = fluoromethylcholine; Chol. = choline; PBS = phosphate-buffered saline.

**FIGURE 5:**
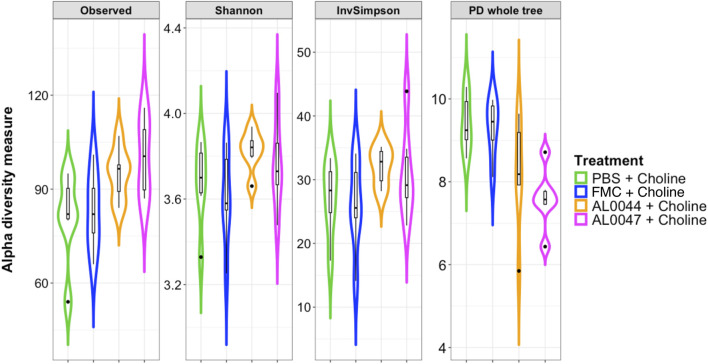
Violin plots with box-and-whisker plots showing the comparison of alpha diversity measures between the experimental conditions at T2, at the end of the experiment. Observed = observed species; Phylogenetic_Diversity = phylogenetic diversity whole tree; Shannon = Shannon–Wiener index; InvSimpson = Inverse Simpson’s index. Median, first, third quartiles and the outliers are shown. No significant changes were found between the conditions under study. FMC = fluoromethylcholine; Chol. = choline; PBS = phosphate-buffered saline.

The NGS analysis of the fecal microbiome of C57Bl/6-Fmo3^em1smoc^ mice treated with the two fermentation products, namely, AL0044 and AL0047 (obtained from *A. sativum* or *C. angustifolia* leaves extracts, respectively), was performed at three sampling time points (T0: before antibiotic treatment, T1: after fecal transplantation, and T2: after treatment). The analysis showed that bacteria belonging to the TMA-producing taxa were initially present in the intestines of animals belonging to all experimental groups after fecal microbiota transplant (T1) (Figure 4). The analysis of stool samples of the mice treated with AL0044 or AL0047 after treatment (T2) also showed that bacterial alpha diversity, measured using the observed species, Shannon, Inverse Simpson index, and phylogenetic diversity indices, remained at the same level as in the mice treated with FMC or untreated (PBS) (p-value >0.5) (Figure 5). In addition, bacteria belonging to the TMA-producing genera or families were still present after treatment with the inhibitors (T2), excluding their bactericidal action (Figure 5).

## 4 Discussions

In this study, we demonstrate that a mixture of postbiotics and tyndallized *L. paracasei* LMG-S-26420 effectively reduces TMA production in both *in vitro* (fecal slurry) and *in vivo* (murine TMAU models) systems. The lead postbiotics, AL0044 (garlic extract-derived) and AL0047 (senna leaf-derived), achieved TMA lyase inhibition comparable to that of the synthetic reference inhibitor FMC. Importantly, this inhibition occurred without bactericidal effects on gut microbiota, as confirmed by preserved alpha diversity and the persistence of TMA-producing genera such as Collinsella and *Clostridium* post-treatment. These findings support a mechanism-based inhibition of TMA lyase while avoiding the dysbiosis risks typically associated with antibiotics. Moreover, the combination of tyndallized biomass and postbiotic supernatant enhanced inhibitory activity, which is consistent with evidence that structural components of inactivated bacteria (e.g., cell wall fragments and metabolites) exert bioactive effects (Gurunathan et al., 2023). This synergy underscores the advantage of “whole postbiotic” formulations over supernatant-only preparations. Compared to current therapeutic strategies, which rely on restrictive diets, antibiotics, or lactulose, the use of postbiotics represents a safer, more stable, and more scalable option. Conventional approaches often have limited efficacy, non-specific microbiota disruption, and poor long-term adherence, while synthetic TMA lyase inhibitors such as FMC or DMB, though promising, face challenges in safety, stability, and scalability (Wang et al., 2015). In contrast, postbiotics offer multiple advantages: safety is ensured by the absence of viable bacteria, eliminating the risk of infection or gene transfer; stability is maintained as lyophilized preparations retain activity without refrigeration, facilitating clinical and commercial use; and scalability is achieved through the optimized process C (heat-inactivated biomass + supernatant), which reduces production costs by 40% compared to that of traditional probiotic methods (data not shown) while simplifying biomass disposal. The broader relevance of these findings extends beyond TMAU, since elevated TMAO is a well-established risk factor for atherosclerosis, thrombosis, and major adverse cardiac events (Koeth et al., 2013; Tang et al., 2013; Latif et al., 2025). By simultaneously reducing malodorous TMA excretion and lowering systemic TMAO, these postbiotics hold potential for dual benefit: symptom relief in TMAU patients and cardiovascular risk mitigation. This is in line with the anti-atherosclerotic effects attributed to garlic-derived allicin, but with the added advantage of standardized industrial production that ensures reproducibility and scalability. Methodologically, this study also introduced several innovations that strengthen its translational relevance. The modified fecal slurry protocol using a poor ingredients medium enabled sensitive TMA quantification while minimizing volatile loss, while NGS confirmed the persistence of TMA-producing taxa. *In vivo*, the use of FMO3-KO mice colonized with humanized microbiota via fecal transplant provided a physiologically relevant model for human TMA metabolism. Analytical rigor was ensured through LC-MS/MS with isotopically labeled standards, enabling precise quantification of TMA and TMAO across biofluids (Ufnal et al., 2014). Nonetheless, several limitations should be acknowledged. The solubility of the fermented matrices could be improved, as the particulate tended to agglomerate, creating difficulties during processing and filtration, and some traces of unfermented matrix were occasionally recovered in the final freeze-dried product. Moreover, large fermentation volumes were required to obtain consistent powder yields. While murine models are highly informative, human clinical trials remain essential to validate the efficacy, dosing, and safety in TMAU and CVD patients. Further mechanistic studies are also needed to isolate and characterize the bioactive molecules responsible for the observed inhibition, for example, through metabolomics and *in vitro* assays with purified TMA lyase (CutC/D) to clarify inhibition kinetics. In addition, longer-term studies beyond 12 days would be valuable to assess the durability of TMA suppression and microbiota stability. Finally, future research should explore broader applications of these postbiotics in other TMAO-linked conditions such as chronic kidney disease.

## 5 Conclusion

In this study, we demonstrate that tyndallized *L. paracasei* LMG-S-26420 combined with garlic- or senna-derived postbiotics (AL0044, AL0047) effectively inhibits intestinal TMA lyase activity in both *in vitro* and *in vivo* models. Critically, these formulations achieved TMA reduction comparable to that of the synthetic inhibitor (FMC) without compromising gut microbiota diversity or eliminating TMA-producing taxa—supporting a targeted, mechanism-based action. The optimized production process (process C) enhanced industrial scalability, reducing costs by 40% while ensuring stability via lyophilization. These postbiotics address key limitations of current TMAU therapies: they circumvent the safety risks of live probiotics, avoid dysbiosis from antibiotics, and outperform dietary restrictions in sustainability. Beyond TMAU, their ability to suppress systemic TMAO positions them as promising candidates for cardiovascular risk mitigation. Patents WO2023/061978A1 (Giannini et al., 2024) and WO2024/209109 (Giannini and Barone, 2024) protect these innovations. Future clinical trials should validate efficacy in human populations and explore applications in other TMAO-associated disorders.

## Data Availability

The data presented in the study are deposited in the NCBI SRA (Sequence Read Archive) repository, accession number PRJNA1200926. Further inquiries can be directed to the corresponding author.

## Abbreviations

TMAU, trimethylaminuria; TMA, trimethylamine; TMAO, trimethylamine N‐oxide; CVD, cardiovascular disease; ASCVD, atherosclerotic cardiovascular disease; GBB, γ-butyrobetaine; FMO, flavin-containing mono-oxygenases; FMO3, flavin-containing mono-oxygenase 3; ISAPP, International Scientific Association for Probiotics and Prebiotics; FMC, fluoromethylcholine; LPS, lipopolysaccharide; FBS, fetal bovine serum; ESI, electrospray ionization; PBS, phosphate-buffered saline; ASVs, amplicon sequence variants; IDE, integrated development environment; LDA, linear discriminant analysis; DMB, 3,3-dimethyl-1-butanol; LM-MS/MS, liquid chromatography–mass spectrometry; HPLC-MS, high-performance liquid chromatography–mass spectrometry.
