# Supplementary material for: A mixture of postbiotics/tyndallized probiotics reduces trimethylamine (TMA) in trimethylaminuria models: Evidence from in vitro and in vivo studies
Source: Front Pharmacol. 2025 Oct 8;16:1591825. doi: 10.3389/fphar.2025.1591825 (PMC12540306; doi:10.3389/fphar.2025.1591825)
Supplement: Supplementary file 1 [file Table1.docx]

**Supplementary table 1.** Fermentation process applied with the relative postbiotics obtained. Process A (Grown biomass – then add matrix. Fermentation in two steps), Process B (Grown biomass – then add matrix. Fermentation in one step), Process C1 (Biomass + supernatant heat treated together in one step), Process C2a (Only biomass from C1, after centrifugation in one step), Process C2b (Only supernatant from C1 after centrifugation in one step)

| **Lab code** | **Product** | **Fermentation process** | **Matrix** |  |
| --- | --- | --- | --- | --- |
| AAT-02/M05/A | AL0010 | Process A | M05 |  |
| AAT-03/M05/B |  | Process B |  |  |
| AAT-04/M05/C1 | AL0044 | Process C1 |  |  |
| AAT-05/M05/C2a |  | Process C2a |  |  |
| AAT-06/M05/C2b |  | Process C2b |  |  |
| AAT-07/M06/A | AL0011 | Process A | M06 |  |
| AAT-08/M06/B |  | Process B |  |  |
| AAT-09/M06/C1 | AL0045 | Process C1 |  |  |
| AAT-10/M06/C2a |  | Process C2a |  |  |
| AAT-11/M06/C2b |  | Process C2b |  |  |
| AAT-12/M09/A | AL0012 | Process A | M09 |  |
| AAT-13/M09/Ref |  | Process B |  |  |
| AAT-14/M09/C1 | AL0046 | Process C1 |  |  |
| AAT-15/M09/C2a |  | Process C2a |  |  |
| AAT-16/M09/C2b |  | Process C2b |  |  |
| AAT-17/M11/A | AL0013 | Process A | M11 |  |
| AAT-18/M11/B |  | Process B |  |  |
| AAT-19/M11/C1 | AL0047 | Process C1 |  |  |
| AAT-20/M11/C2a |  | Process C2a |  |  |
| AAT-21/M11/C2b |  | Process C2b |  |  |

**Supplementary table** **2.** Mouse groups with relative compounds concentration administered

| **Groups** | **mouse** | **diet** | **Compounds** | **Concentration** |
| --- | --- | --- | --- | --- |
| 1 | 6 | Choline | *Allium Sativum* (M05) | 2 mg/mouse/daily |
| 2 | 6 | Choline | (AL0044)  AAT-04/M05/C1 | 2 mg/ mouse /daily |
| 3 | 6 | Choline | (AL0010)  AAT-02/M05/A | 2 mg/ mouse /daily |
| 4 | 6 | Choline | leaves of *Cassia angustifolia* (M11) | 2 mg/ mouse /daily |
| 5 | 6 | Choline | *(AL0047)*  AAT-19/M11/C1 | 4 mg/ mouse /daily |
| 6 | 6 | Choline | (AL0013)  AAT-17/M11/A | 4 mg/ mouse /daily |
| 7 | 6 | Choline | FMC (Positive control) | 2 mg/ mouse /daily |
| 8 | 6 | Choline | PBS (negative control) | 0,2 ml/ mouse /daily |

**Supplementary table 3.** Subdivision of mice groups, compounds to be tested and their concentrations

| **2st**  **replicate** | **Groups** | **Mouse** | **Diet** | **Compounds** | **Concentration** |
| --- | --- | --- | --- | --- | --- |
|  | 1 | 3 | Choline | PBS (negative control) | 0,2 ml/mouse/daily |
|  | 2 | 7 | Choline | AL0044  AAT-04/M05-C1 | 2 mg/mouse/daily |
|  | 3 | 7 | Choline | AL0047  AAT-19/M11-C1 | 4 mg/mouse/daily |
|  | 4 | 3 | Choline | FMC  (positive control) | 2 mg/mouse/daily |
